# Plasma arachidonic and docosahexaenoic acids in Tunisian very low birth weight infants: status and association with selected neonatal morbidities

**DOI:** 10.1186/s41043-015-0011-3

**Published:** 2015-06-24

**Authors:** Samira Fares, Mohamed M. Sethom, Samia Kacem, Chahnez Khouaja-Mokrani, Moncef Feki, Naziha Kaabachi

**Affiliations:** 1 UR05/08-08, Department of Biochemistry, Rabta Hospital & Faculty of Medicine of Tunis, El Manar University, Tunis, Jebbari 1007 Tunisia; 2 Service of Neonatalogy, Centre of Maternity and Neonatology & Faculty of Medicine of Tunis, El Manar University, 1007 Tunis, Tunisia

**Keywords:** Arachidonic acid, Docosahexaenoic acid, Intraventricular hemorrhage, Sepsis, Preterm infants, Respiratory distress syndrome, Small for gestational age

## Abstract

To study plasma arachidonic acid (AA) and docosahexaenoic acid (DHA) status in Tunisian very low birth weight (VLBW) infants and their association with selected neonatal morbidities. A total of 709 VLBW infants and 339 term infants were included. Plasma fatty acids were analyzed using capillary gas chromatography. VLBW infants had significantly (p < 0.001) lower plasma AA (9.44 ± 2.12 *vs.* 10.8 ± 2.10) and DHA (2.56 ± 0.89 *vs.* 3.46 ± 1.09) levels, but higher n-6:n-3 ratio (5.58 ± 1.22 *vs.* 5.17 ± 1.46) than term infants. In VLBW infants, plasma AA and DHA were related to gestational age (r = 0.156; p = 0.001 and r = 0.134; p = 0.003, respectively), birthweight (r = 0.242; p < 0.001 and r = 0.181; p < 0.001, respectively) and head circumference (r = 0.138; p = 0.005 and r = 0.108; p = 0.027, respectively). Infants with respiratory distress syndrome have decreased plasma AA and DHA and those with intraventricular hemorrhage have decreased plasma AA and n-6:n-3 ratio. Sepsis was associated with decreased DHA levels. Plasma long chain polyunsaturated fatty acids status is low in VLBW infants. These deficits may enhance the risk of common neonatal morbidities, rendering their prevention and correction greatly warranted.

## Background

Long chain-polyunsaturated fatty acids (LCPUFAs) arachidonic acid (AA, C20:4 n-6) and docosahexaenoic acid (DHA, C22:6 n-3) are critical for neural, visual and vascular development [1, 2]. The development and maturation of the nervous system in Humans begins in utero and extends over the two first years of life, period during which the needs of the fetus and newborn in AA and DHA are elevated [3, 4]. Fetal needs in these LC-PUFAs are mainly covered by placenta transfer, which substantially increases during the third trimester of pregnancy with little synthesized into fetus [1, 5]. Preterms may be in disadvantage compared to term infants regarding PUFAs status due to a shortened gestation [4, 6] and low activity of enzymes responsible of endogen synthesis of LCPUFAs [7–9]. In addition to their role in neurological and vascular development, AA and DHA are essential modulators of immune function and inflammation. Their alteration during the postnatal period in premature infants may results in a dysregulation of immune and inflammatory responses, which may predispose them to neonatal morbidities [10–12]. Thus, the evaluation of PUFAs status at birth could be an important predictor for infants' development and health. The present study was aimed to determine the AA and DHA status and its determinants in VLBW infants and to test their association with selected neonatal morbidities.

## Methods

### Subjects

The study included 736 preterm VLBW neonates (birth weight <1500 g and gestational age <37 weeks) and 339 term healthy neonates (birth weight between 2500 and 3500 g) as controls. All neonates were born between 2005 and 2008 in The Center of Maternity and Neonatology of Tunis. This Center is the most important public maternity hospital in Great Tunis region and draws pregnant women of low to average socioeconomic rank. Malformed neonates, those with chromosomal abnormality were excluded. Infants with birth weight <650 g or gestational age <27 weeks were not included as their chances of survival are greatly reduced in our practice. The study protocol was approved by the Ethics Committee of Maternity Center and informed consent was obtained from each mother.

### Maternal and infant characteristics

Relevant information was collected from medical records. They included maternal age, medical and obstetrical history and the course of the current pregnancy. Almost all mothers had prenatal care. The majority of them have low fish intake (average once a month) and no one has taken fish oil or vitamins supplements during the pregnancy. Preeclampsia and gestational diabetes are defined according to American College of Obstetricians and Gynecologists criteria [13, 14]. Infant's data included gender, gestational age, birthweight and existence of small for gestational age (SGA), as well as the occurrence during the hospital stay of selected neonatal diseases including respiratory distress syndrome (RDS), sepsis and intraventricular hemorrhage (IVH). SGA is defined as a weight below the 10^th^ percentile for the gestational age [15]. RDS was defined as respiratory distress with CXR abnormality, requiring respiratory support in the form of nasal continuous positive airway pressure or mechanical ventilation or the administration of surfactant. Late-onset sepsis was defined as the occurrence of at least one episode of clinical symptoms of infection and a positive result on 1 or more blood cultures obtained after 72 h of life. Diagnosis of IVH was based on specific features in serial transfontanellar ultrasound [16]. For reason of information’s lack in some records, the association of fatty acids with neonatal morbidities was studied in 480 preterm infants.

### Collection of blood samples and preparation

Blood was collected following medical prescription for cell count analysis in VLBW infants and for ABO Rhesus grouping in term infants. Blood (1 to 2 ml) was drawn between 8 and 10 a.m. by veinupuncture from neonates within the first 24 h of life into EDTA containing tubes. After completion of the prescribed analysis, the tube was recovered (into 2 h) and centrifuged at 2500 g. Plasma (300 μl) was added with 20 μL butylated hydroxytoluene (25 mg in 100 ml ethanol) as antioxidant and stored at −80 °C until analysis (within 6 months).

### Analytical methods

Plasma fatty acids were analyzed by capillary gas chromatography according to the method of Moser and Moser [17]. Plasma lipids were extracted by methylene chloride/methanol mixture in presence of heptadecanoic acid as internal standard and hydrolyzed by potassium carbonate, and fatty acids were methylated in presence of acetyl chloride. The resulting fatty acid methyl esters were extracted by hexane and analyzed with a gas chromatograph model 6890 N (Agilent Technologies, Santa Clara, CA), equipped with split/splitless capillary intel system and flame ionization detector. Separation was achieved on capillary column (Innowax; 30 m × 0.25 mm; ID, 0.25 mm; Agilent Technologies) using nitrogen as carrier gas. The oven temperature was programmed from 150 °C to 250 °C. The injector and detector temperatures were 230 and 280 °C, respectively. The fatty acids were identified by comparison of relative retention time with authentic standards and results are expressed as percent of total fatty acids weight (mol%).

### Statistical analysis

Statistical analysis was performed using the SPSS version 15.0 software package (SPSS Inc., Chicago, USA). The data of each continuous variable were examined for normality using the Kolmogrov-Smiranov test. Continuous variables were compared between groups using independent-samples *T* test. The relationship between continuous variables was tested using Pearson r coefficient of correlation. In order to test how the association between fatty acids and selected neonatal illnesses is independent of confounding factors, multi linear regression models were performed with AA, DHA or n-6:n-3 ratio as response variable, and gestational age, birthweigt, twin pregnancy, preeclampsia, gestational diabetes and selected neonatal illness (SGA, RDS, sepsis or IVH) as independent variables. Goodness-of-fit of logistic models were satisfactory. A p value < 0.05 based on two-sided calculation was considered significant.

## Results

The main maternal and preterm infants’ characteristics and neonatal outcomes are shown in Table 1. Compared to term infants, VLBW infants showed significantly higher plasma saturated fatty acids (SFAs) and monounsaturated fatty acids (MUFAs), but lower PUFAs. Both plasma AA and DHA were lower, and n-6:n-3 ratio was significantly higher in VLBW compared to term infants. The differences remained significant when excluding infants whose mothers have suffered from preeclampsia or gestational diabetes (Table 2).

**Table 1:** Data of preterm infants and mothers

	Preterm infants (n = 480)
Infant's characteristics	
Gestational age, weeks	
27–31	340 (70.8 %)
32–37	140 (29.2 %)
Birthweight, g	
650–999	107 (21.1 %)
1000–1499	373 (77.9 %)
Gender	
Male	240 (50.0 %)
Female	240 (50.0 %)
Maternal and pregnancy characteristics	
Twin pregnancy	149 (31.0 %)
Preeclampsia	214 (44.6 %)
Gestational diabetes	24 (5.0 %)
Cesarean section	338 (70.4 %)
Neonatal morbidities	
Small for gestational age	142 (29.6 %)
Respiratory distress syndrome	234 (48.8 %)
Sepsis	218 (45.4 %)
Intaventricular hemorrhage	86 (17.9 %)

Values represent number of cases (percent)

**Table 2:** Plasma fatty acid profile (mol%) at birth in term and preterm infants

	Term infants	Preterm infants	
	(n = 339)	All (n = 709)	Without GD or PE in mothers (n = 482)
Palmitic acid (C16:0)	30.0 (2.56)	30.5 (2.25)**	30.6 (2.29)**
Stearic acid (C18:0)	10.0 (1.62)	10.7 (1.54)***	10.7 (1.53)***
Oleic acid (C18:1 n-9)	24.8 (2.57)	27.2 (2.11)***	27.3 (2.01)***
Linoleic acid (C18:2 n-6)	10.7 (1.99)	9.37 (2.14)***	9.27 (2.14)***
Alpha Linolenic acid (C18:3 n-3)	0.39 (0.17)	0.31 (0.12)***	0.30 (0.12)***
Arachidonic acid (C20:4 n-6)	10.8 (2.10)	9.44 (2.12)***	9.35 (2.09)***
Eicosapentaenoic acid (C20:5 n-3)	0.54 (0.32)	0.52 (0.30)**	0.48 (0.30)**
Docosahexaenoic acid (C22:6 n-3)	3.46 (1.09)	2.56 (0.89)***	2.55 (0.88)***
Saturated fatty acids (SFAs)	41.2 (3.29)	42.7 (3.20)***	42.8 (3.25)***
Monounsaturated fatty acids (MUFAs)	29.9 (2.90)	32.7 (2.56)***	32.8 (2.47)***
Polyunsaturated fatty acids (PUFAs)	28.8 (4.39)	24.7 (5.01)***	24.5 (4.96)***
Essential fatty acids (EFAs)	11.1 (2.03)	9.71 (2.24)***	9.61 (2.24)***
n-6:n-3 PUFAs	5.17 (1.46)	5.58 (1.22)***	5.51 (1.16)***

*GD* gestational diabetes, *PE* preeclampsia; SFAs = C14:0 + C16:0 + C18:0; MUFAs = C16:1 n-7 + C18:1 n-9; EFAs = C18:2 n-6 + C18:3 n-3; PUFAs = n-6 PUFAs (C18:2 n-6 + C18:3 n-6 + C20:3 n-6 + C20:4 n-6) + n-3 PUFAs (C18:3 n-3 + C22:5 n-3 + C22:6 n-3)

Values represent mean (SD); **, p < 0.01, ***, p < 0.001 (compared to term infants)

**Fig. 1:**
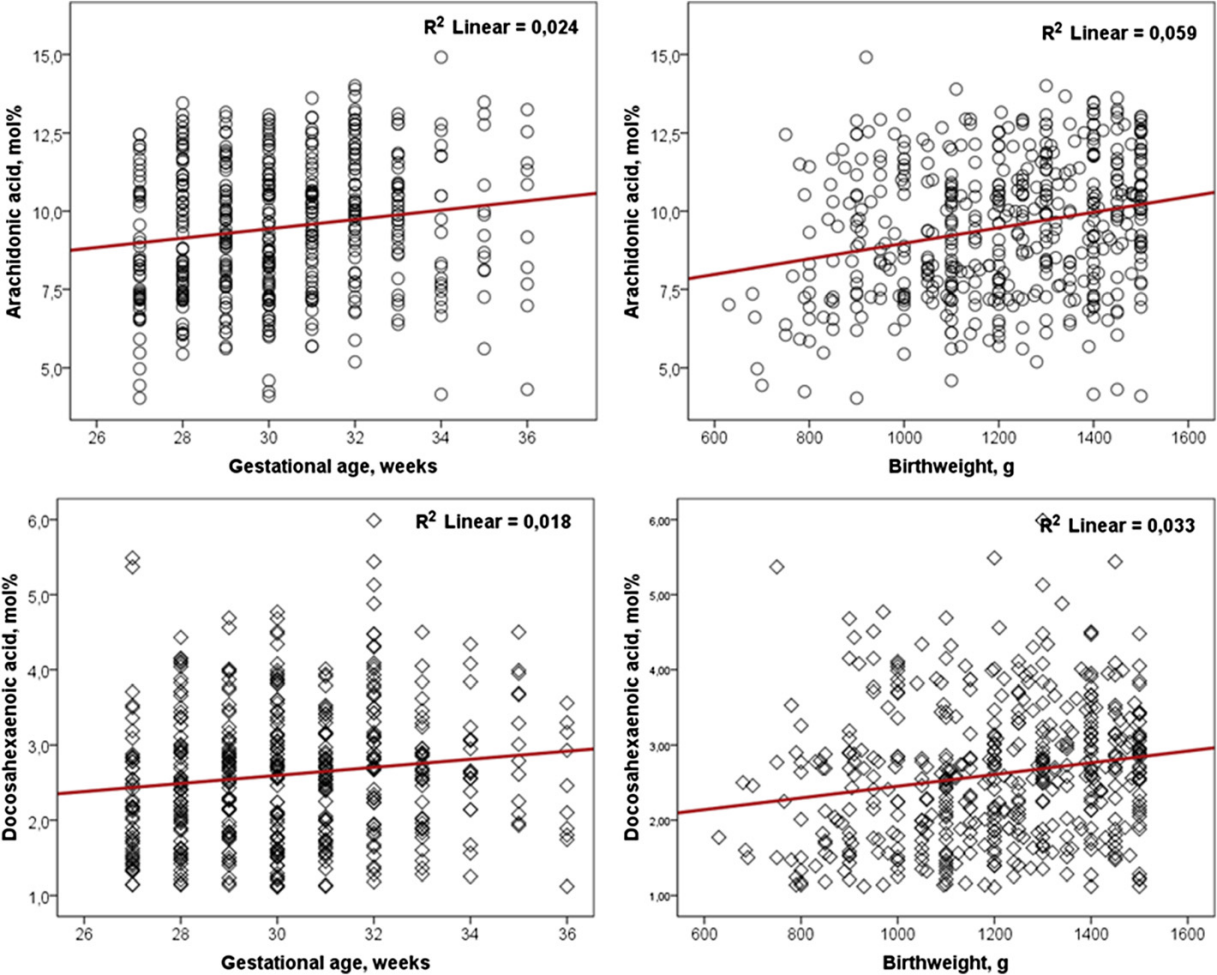
Correlations of plasma arachidonic acid and docosahexaenoic acid with gestational age and birthweight (n = 480)

In VLBW infants, no gender differences were observed for either individual fatty acids or n-6:n-3 ratio. Infants issued from twin pregnancy have a significant lower plasma DHA (2.44 ± 0.84 *vs.* 2.68 ± 0.92, p = 0.007) and higher n-6:n-3 ratio (5.78 ± 1.17 *vs.* 5.45 ± 1.28; p = 0.009) than singleton. Plasma AA and DHA, but not n-6:n-3 ratio were correlated with gestational age (r = 0.156, p = 0.001 for AA and r = 0.134, p = 0.003 for DHA), birthweight (r = 0.242, p < 0.001 for AA and r = 0.181, p < 0.001 for DHA) (Fig. 1), and head circumference (r = 0.138, p = 0.005 for AA and r = 0.108, p = 0.027 for DHA).

No differences were observed for plasma AA, DHA levels and n-6:n-3 ratio according to SGA. In univariate analysis, VLBW infants who developed sepsis had lower plasma DHA levels and those with RDS have significantly lower plasma AA levels. Infants with IVH had lower AA levels and n-6:n-3 ratio. In multi linear regression models, AA was related to birthweight, DHA was related to birthweight, twin pregnancy and RDS, and n-6:n-3 ratio was related to twin pregnancy and IVH (Table 3).

**Table 3:** Plasma arachidonic acid (AA) and docosahexaenoic acid (DHA) levels (in mol%) and n-6:n-3 ratio in VLBW infants according to selected neonatal morbidities (n = 480)

			AA (mol%)	DHA (mol%)	n-6:n-3 ratio
Small for gestational age	No	338	9.46 (2.10)	2.61 (0.87)	5.53 (1.20)
	Yes	142	9.48 (2.30)	2.60 (0.99)	5.60 (1.38)
Respiratory distress syndrome	No	246	9.71 (2.19)	2.74 (0.95)	5.66 (1.21)
	Yes	234	9.22 (2.09)**	2.47 (0.91)***^,^****	5.44 (1.28)*
Sepsis	No	262	9.60 (2.13)	2.69 (0.94)	5.52 (1.31)
	Yes	218	9.32 (2.18)	2.51 (0.85)*	5.59 (1.20)
Intraventricular hemorrhage	No	394	9.55 (2.11)	2.62 (0.93)	5.62 (1.27)
	Yes	86	9.01 (2.20)*	2.56 (0.82)	5.27 (1.22)*^,^****

Values are expressed as mean (SD); *, p < 0.05; **, p <0.01; ***, p < 0.001 (univariate analysis); ****, p < 0.05 (multivariate analysis, adjusting for gestational age, birthweight, twin pregnancy, preeclampsia and gestational diabetes)

## Discussion

This study showed lower plasma AA and DHA levels in Tunisian VLBW neonates compared to term infants. In VLBW neonates, AA and DHA levels were associated with the degree of prematurity; the lower the gestational age and the birthweight the lower AA and DHA levels.

The delivery of PUFAs substantially increases during the third trimester, coinciding with continued organ development and rapid fetal growth [5, 17]. Fatty acid placental transfer is characterized by the biomagnification phenomenon, consisting in preferential placental delivery of DHA and AA to the fetus [18]. Preterm delivery interrupts placental supply of these critical fatty acids and prevents the effect of biomagnification. The early termination of selective fatty acid delivery, coupled with a feeble LCPUFAs synthesis from fatty acid precursors and a lack of adipose tissue stores in immature tissues [3, 9] may explain the low DHA and AA status in VLBW infants. During neonatal life, PUFA needs are greater in preterm infant who requires more nutrients to ensure maturation and development of its tissues and organs. However, the nutritional management strategies usually fail to meet the LCPUFA fetal accretion requirements and thus may contribute to further decline of these fatty acids during the first postnatal period [18–20]. As a consequence, preterm infants are disadvantaged with respect to access to AA and DHA needed for brain maturation compared with term infants. These deficits would expose VLBW infants to a higher risk for neonatal morbidities [20]. Gestational diabetes and preeclampsia are known to limit placental transfer of PUFAs. These conditions are very common in our series and would have contributed to the altered fatty acids profile in VLBW infants. However, plasma AA and DHA remained low when infants issued from pregnancies with these conditions were excluded.

The study showed that plasma AA and DHA levels are lower in VLBW neonates who will develop RDS or sepsis. These fatty acids and their derivatives have ability to modulate immune function and inflammatory responses. Their alteration during the postnatal period in premature infants may contribute to a dysregulation of immune and inflammatory responses, which may predispose them to neonatal morbidities. DHA-derived metabolites such as resolvins decrease neutrophil infiltration and enhance macrophage phagocytosis [21]. DHA also down regulates nuclear factor κB (NF-κB) activity in cells either directly or via increased activation of peroxisome proliferator activated receptors (PPARs), thereby reducing cytokine release [22, 23]. Lastly, DHA competes with AA for incorporation into cell membranes thus limiting the proinflammatory signaling mediated by AA [21, 23]. Therefore, low levels of DHA would be expected to predispose to increased host inflammatory responses such as that seen in sepsis. In RDS, decreased AA levels may increase the risk by inhibiting the innate immune response through decreased eicosanoids, in particular, leukotrienes, which are known to enhance chemotaxis of leukocytes, neutrophil activation, and activity of natural killer cells [22].

In these preterm infants, low plasma AA levels were associated with a greater risk for IVH. This bleeding into the microvascular tissue lining the brain ventricles involves factors that affect cerebral vascular tone and flux, angiogenesis, inflammation and coagulation [24]. Reduced AA levels could lead to leaking cell membranes and impair the balance of vasodilator and vasoconstrictor and pro- and antithrombotic eicosanoids produced by the endothelium and platelets [1, 25].

The n-6:n-3 ratio has functional consequences in addition to individual fatty acids in influencing disease [26, 27]. This ratio regulates inflammatory mediators and other downstream modulators of cell and organ physiology [26–28]. Our data showed that n-6:n-3 ratio is decreased in infants with IVH. This decrease is due to low rates of n-6 PUFAs, mainly LA and AA. Previous studies have showed that a better control of the n-6:n-3 balance may represent an interesting target in the prevention and/or control of a large number of neonatal morbidities in premature infants, such as sepsis and chronic diseases.

## Conclusion

In total, LCPUFAs, mainly AA and DHA are low in VLBW Tunisian neonates. Such decrease is related to lack in transplacental transfer and reduced activity of enzymes responsible for endogen synthesis from essential fatty acids. Altered fatty acid levels may favor the development of common neonatal morbidities such as RDS, sepsis and IVH in these neonates. Therefore, efforts should be undertaken to enhance the PUFAs status in preterm infants. The strategy would include an increase of LCPUFAs intake in pregnant and breastfeeding women and feeding neonates with LCPUFAs-rich formula or LCPUFAs-fortified breast milk. Tight control of recurrent infections, preeclampsia and gestational diabetes in pregnant women may fight against premature birth and then prevent LCPUFAs deficits in neonates.
